# Genotypic profiles of *Leishmania (Viannia) braziliensis* strains from cutaneous leishmaniasis patients and their relationship with the response to meglumine antimoniate treatment: a pilot study

**DOI:** 10.1051/parasite/2017035

**Published:** 2017-09-29

**Authors:** Thalita Gagini, Armando de Oliveira Schubach, Maria de Fatima Madeira, Cláudia Maria Valete-Rosalino, Maria Inês Fernandes Pimentel, Raquel da Silva Pacheco

**Affiliations:** 1 Laboratório de Pesquisa Clínica e Vigilância em Leishmanioses, Instituto Nacional de Infectologia Evandro Chagas INI/FIOCRUZ, Manguinhos, Rio de Janeiro Brazil; 2 Departamento de Otorrinolaringologia e Oftalmologia, Faculdade de Medicina, UFRJ, Hospital Universitário Clementino Fraga Filho, Cidade Universitária - Ilha do Fundão, Rio de Janeiro Brazil

**Keywords:** Cutaneous leishmaniasis, genetic heterogeneity, *Leishmania (Viannia) braziliensis*, LSSP-PCR

## Abstract

*Background:* Forty-four strains isolated from a cohort of cutaneous leishmaniasis (CL) patients who did or did not respond to one course of treatment with meglumine antimoniate were investigated to explore genetic polymorphisms in parasite kinetoplast DNA minicircles. *Leishmania (Viannia) braziliensis* strains isolated from responder (R) and non-responder (NR) patients who acquired infection in Rio de Janeiro or in other Brazilian states were studied using low-stringency single-specific primer polymerase chain reaction (LSSP-PCR) to identify genetic polymorphisms. *Results:* Polymorphisms were observed in parasites recovered from patient lesions. No association was found between a specific genotype and R or NR patients. Phenetic analysis grouped the genotypes into three main clusters, with similarity indices varying from 0.72 to 1.00. Although no specific genotype association was detected, at least one group of *L. (V.) braziliensis* genotypes that circulates in Rio de Janeiro was discriminated in clusters I and III, showing phenotypes of good and poor responses to treatment, respectively. Cluster I comprised parasite profiles recovered from R patients from Rio de Janeiro and in cluster III, NR samples were prevalent. Cluster II comprised 24 isolates, with 21 from Rio de Janeiro and three from other states, equally distributed between R and NR patients. Additionally, we found that parasites sharing all common genetic characteristics acted differently in response to treatment. *Conclusions:* These results are of clinical-epidemiological importance since they demonstrate that populations of *L. (V.) braziliensis* that exhibit high levels of genetic similarity also display different phenotypes associated with meglumine antimoniate responses in cutaneous leishmaniasis patients.

## Abstract (fr)

*Contexte :* Quarante-quatre souches isolées d'une cohorte de patients atteints de leishmaniose cutanée (CL) qui ont répondu ou non à un schéma de traitement avec de l'antimoniate de méglumine ont été étudiées pour explorer le polymorphisme génétique dans les minicerles d'ADN des kinétoplastes des parasites. Les souches de *Leishmania (Viannia) braziliensis* isolées des patients répondeurs (R) et non-répondeurs (NR) qui ont subi une infection à Rio de Janeiro ou dans d'autres états brésiliens ont été étudiées en utilisant une réaction en chaîne à la polymérase spécifique à faible stringence (LSSP-PCR) pour identifier le polymorphisme génétique. *Résultats :* Du polymorphisme a été observé chez les parasites récupérés dans les lésions de patients. Aucune association n'a été trouvée entre un génotype spécifique et les patients R ou NR. L'analyse phénétique a regroupé les génotypes en trois groupes principaux, avec des indices de similarité variant de 0,72 à 1,00. Bien qu'aucune association de génotype spécifique n'ait été détectée, au moins un groupe de génotypes de *L. (V.) braziliensis* qui circule à Rio de Janeiro a été discriminé dans les groupes I et III, montrant respectivement des phénotypes de bonnes et de mauvaises réponses au traitement. Le groupe I comprenait des profils de parasites récupérés chez des patients R de Rio de Janeiro et dans le groupe III, les échantillons NR étaient fréquents. Le groupe II comprenait 24 isolats, dont 21 de Rio de Janeiro et trois d'autres états, répartis de façon égale entre les patients R et NR. En outre, nous avons constaté que les parasites partageant toutes les caractéristiques génétiques communes agissaient différemment en réponse au traitement. *Conclusions :* Ces résultats sont d'importance clinico-épidémiologique, par la démonstration que les populations de *L. (V.) braziliensis* qui présentent des niveaux élevés de similarité génétique présentent également différents phénotypes associés aux patients atteints de leishmaniose cutanée et leur réponse à l'antimoniate de méglumine.

## Introduction

Leishmaniases are a group of zoonotic or anthroponotic diseases caused by different species of the genus *Leishmania*. The diseases occur in different environments and are endemic in 98 countries and three territories on five continents, affecting more than 12 million people worldwide. Tegumentary leishmaniasis (TL) is widely distributed, with approximately 0.7 million to 1.2 million TL cases diagnosed annually [1]. TL presents as two distinct clinical forms: cutaneous and mucosal, depending on the species of parasite involved in the infection and the immune response of the host. Cutaneous leishmaniasis (CL) comprises clinical manifestations occurring exclusively on the skin [2].

Meglumine antimoniate is the drug of choice for CL treatment due to its low cost and efficacy; however, adverse reactions are often reported resulting from its high toxicity (antimonial doses should not exceed 20 mg Sb^5+^/kg/day) [3]. In Brazil, the Ministry of Health [2] recommends 10 to 20 mg Sb^5+^/kg/day for 20 consecutive days, not exceeding 1215 mg Sb^5+^ per day. The efficacy of this regimen in regions where *Leishmania (Viannia) braziliensis* infections predominate varies between 51.1% and 90%. In Brazil, therapeutic failure defined as a lack of clinical cure after receiving two regular therapeutic courses varies from 46 to 75% [2,4,5]. However, antimony resistance is not a national public health concern, and from 50 to 100% of re-treated patients have a favorable outcome [2].

At the Evandro Chagas National Institute of Infectious Diseases, Oswaldo Cruz Foundation (INI/FIOCRUZ), Rio de Janeiro, Brazil, a therapeutic regimen comprising 5 mg Sb^5+^/kg/day given intramuscularly is used, with results close to those obtained in patients treated with 20 mg Sb^5+^/kg/day, and this regiment is associated with a low rate of therapeutic failure (14–22.2%) after a single course of meglumine antimoniate [6]. However, at the INI, we generally treat patients who have a poor initial therapeutic response or who relapse after an apparently favorable initial therapeutic response with 5 mg Sb^5+^/kg/day or intralesional meglumine antimoniate once or twice, before attempting another drug. Therefore, of the patients who were originally treated with low-dose antimony and who were followed up after clinical failure, 85.7% or more were cured after one or two additional treatments with either intralesional or low-dose meglumine antimoniate. Some patients re-evaluated up to 14 years after treatment remained clinically cured [7]. A hypothesis has been put forward suggesting that the therapeutic success of 5 mg Sb^5+^/kg/day observed in patients from the state of Rio de Janeiro may be attributed to the genetic characteristics of the circulating strains of *L. (V.) braziliensis* in this region, which may be associated with high susceptibility to meglumine antimoniate.

High genetic diversity among *Leishmania* parasites, especially *L. (V.) braziliensis*, which is the most prevalent species in Brazil, has been reported [8,9]. Different epidemiological patterns may influence the levels of genetic variability to as great a degree as the influence of parasitic population diversity on the evolution and distribution of virulence-related characteristics, drug resistance, and infectivity [10]. The technique involving a low-stringency single-specific primer polymerase chain reaction (LSSP-PCR) [11] used to detect sequence divergences in kinetoplast DNA (kDNA) minicircles has proven to be a valuable tool for distinguishing genetic heterogeneity in parasite populations at the intraspecific and interspecific levels [12,13]. Differences in prevalent classes of kDNA minicircles have been applied to define and associate specific *Leishmania* genotypes with biological attributes or clinical conditions in infected patients [8,12,14].

We carried out a pilot exploratory study to evaluate the genetic polymorphisms in *L. (V.) braziliensis* kDNA minicircles based on samples from a cohort of cutaneous leishmaniasis patients classified as responders (R) or non-responders (NR) to one course of meglumine antimoniate treatment.

## Materials and methods

### Ethics

This study was approved by the Ethics in Research Committee (CEP/INI; No. 0065.0.009.000-11), and all patients voluntarily signed an informed consent form to authorise sample storage.

### Research participants and samples

An observational study was conducted in a cohort of CL patients monitored at INI, Rio de Janeiro, Brazil, between 1999 and 2011 with reported infections acquired in several states of Brazil, including Rio de Janeiro. Clinical data for the patients were accessed by examining their medical records.

The inclusion criteria were treatment-naive patients with localized CL diagnosis (primary involvement of the skin with one to fewer than 20 ulcerous lesions, usually with good response to treatment) on the basis of clinical signs (suggestive cutaneous lesions), immunological criteria (*Leishmania* serology using indirect immunofluorescence reaction (IIF) and enzyme-linked immunosorbent assay (ELISA)), and parasitological criteria (positive culture for *Leishmania* in suitable media), and patients whose samples were identified as *L. (V.) braziliensis* and preserved in liquid nitrogen. Additionally, patients who received the first treatment for CL at INI with meglumine antimoniate 5 mg Sb^5+^/kg/day, intramuscularly, for 30 consecutive days or with meglumine antimoniate 20 mg Sb^5+^/kg/day, intramuscularly, for 20 consecutive days, were included.

Exclusion criteria were patients with clinical forms other than localized CL, lack of post-treatment follow-up for at least 2 years, or those presenting comorbidities.

### Clinical and laboratory data

Clinical and laboratory data were collected directly from the records of the selected patients. We evaluated gender, age in years, Brazilian state where the infection likely occurred, number of skin lesions, lesion location [cervical-facial region (head), torso, upper limbs, or lower extremities], lesion-evolution time in days, Montenegro skin test (MST) diameter of induration (negative: 0–4 mm; positive: ≥5 mm), ELISA (cut-off point calculated for each reaction based on optical density values), and IIF (cut-off point considered positive ≥1/40).

The populations being studied were classified by therapeutic outcome. The R group included patients who presented clinical healing defined as complete epithelization of the cutaneous lesions up to 3 months after the conclusion of one course of treatment, and subsequent total regression of crusts, desquamation, infiltration, and erythema, without any sign of reactivation and absence of mucosal lesions over a 1 year observation period. The NR group included patients experiencing therapeutic failure (absence of complete skin-lesion epithelization up to 3 months after the end of one treatment) or lesion recurrence (reappearance of skin or mucosal lesions up to 1 year after clinical healing) requiring new courses of treatment.

### Sample preparation

*Leishmania* promastigotes isolated from biopsies of cutaneous lesions were immediately stored in liquid nitrogen and subsequently recovered after two passages in Schneider's *Drosophila* medium (Sigma-Aldrich, St. Louis, MO, USA) supplemented with 10% fetal bovine serum (WL-Inmunoquimica, Rio de Janeiro, BR) at 26 °C. Parasites in the stationary phase were harvested by centrifugation at 4000 rpm for 10 min at 4 °C and washed several times with sterilized phosphate-buffered saline (pH 7.2). The *L. (V.) braziliensis* reference strain MHOM/BR/75/M2903 was used as a control in all experiments.

The isolated parasites were previously characterized as *Leishmania (V.) braziliensis* by multi-locus enzyme electrophoresis (MLEE) analyses according to previously defined protocols [15]. The reference strains *L. (V.) guyanensis* MHOM/BR/1975/M4147 and *L. (L.) amazonensis* IFLA/BR/1967/PH8 were also used in MLEE experiments.

### LSSP-PCR analysis

DNA extraction was performed using the Illustra tissue cells genomic prep mini & spin kit (GE Healthcare, Little Chalfont, UK) according to the manufacturer's instructions. Specific PCR was performed using the primers B1 (5′-GGGGTTGGTGTAATATAGTGG-3′) and B2 (5′-CTAATTGTGCACGGGGAGG-3′) targeting the variable region of kDNA minicircles that amplify a 750 bp product specific to the *L. braziliensis* complex [16]. Purification of the amplified products was performed using the Illustra GFX PCR DNA kit and gel band purification (GE Healthcare). Purified products underwent a second amplification using primer B1 under low-stringency conditions and using high concentrations of Taq DNA polymerase according to the protocol described by Oliveira et al. [14]. LSSP-PCR bands varying from 300 to 750 bp were scored and compared using the simple matching coefficient of similarity to determine the proportion of mismatched bands between pairs of isolates. A total of 14 characteristics (bands) were evaluated (see Table 2 and Figure 2). The binary matrix was constructed considering “zero” for the absence and “1” for the presence of a specific band. The similarity matrix was transformed into a dendrogram using the UPGMA algorithm. Phenetic analysis was performed using the NTSYS-pc program, version 2.02 (Exeter Software, Setauket, NY, USA).

### Statistical analysis

The Statistical Package for the Social Sciences (SPSS) for Windows, version 16.0 (SPSS Inc., Chicago, IL, USA) was used for data analysis. The simple frequencies of the categorical variables were described, as well as the summary measures [mean ± standard deviation (SD), median, minimum and maximum] of the continuous variables. The association between categorical variables with clusters and therapeutic outcome was verified by Pearson's chi-squared test, Fisher's exact test, and the Shapiro-Wilk normality test. The Mann-Whitney *U* test was used to compare the median of number of treatments, MST (mm), number of lesions, and evolution time (days), whereas Student's *t-*test was used to compare mean age by cluster and by therapeutic outcome. A *p* < 0.05 indicated a significant difference.

## Results

### Patient clinical and geographical data

**Figure 1:**
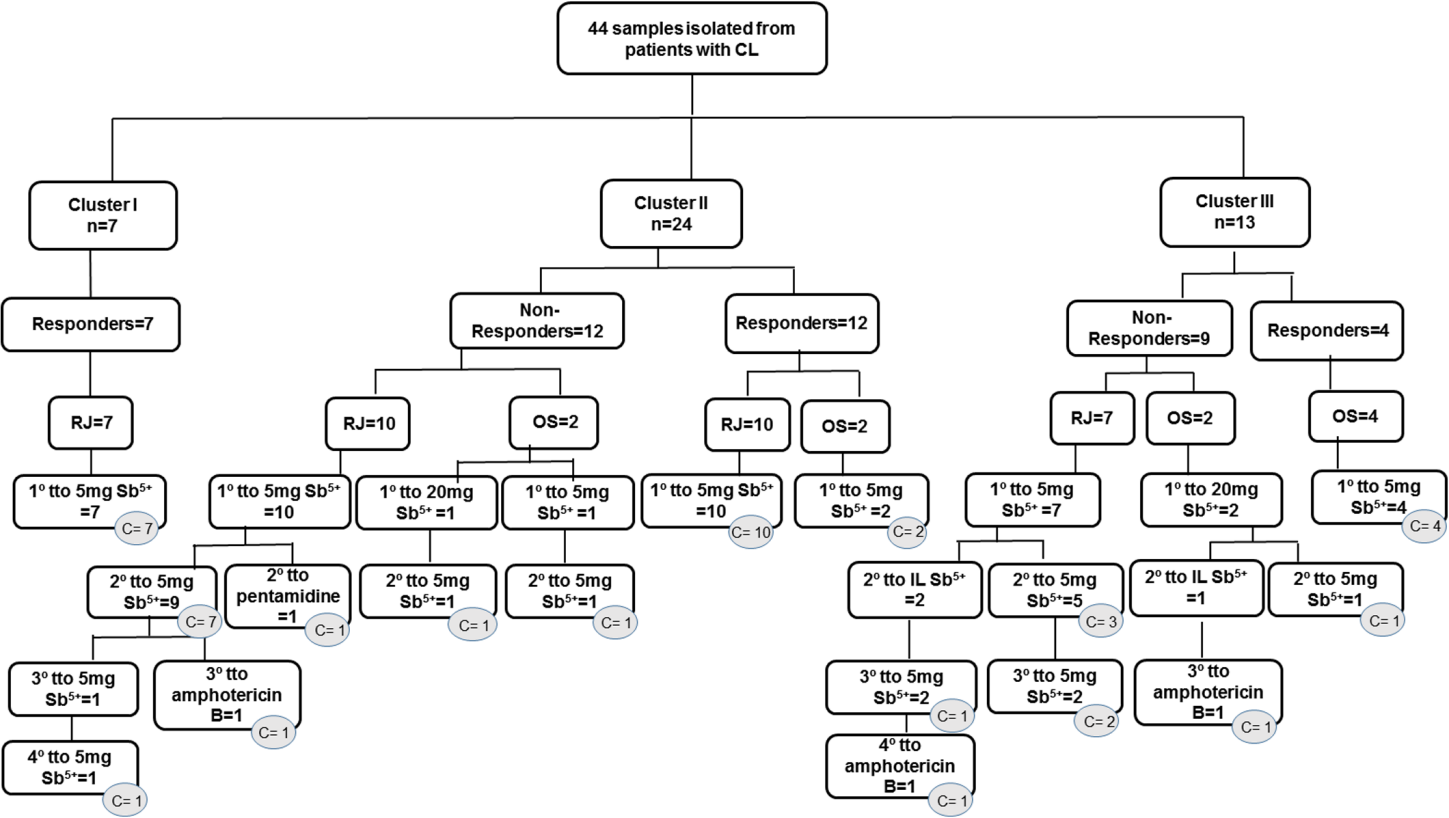
Illustrative flow diagram of treatments administered to groups of 44 cutaneous leishmaniasis patients classified by *Leishmania (Viannia) braziliensis* clusters, by type of therapeutic response to one treatment course and by localization of infection. Circles filled in gray indicate the treatment (one course or additional ones) that promoted patient cure. RJ = Rio de Janeiro; OS = other states; tto = treatment, IL = intralesional; C = cure.

Forty-four patient samples were included in this study, with 26 (59.1%) from men and 18 (40.9%) from women [aged 11–59 years (34.25 ± 12.25)]. Patients harbored from 1 to 15 (median = 1) cutaneous lesions; in 23 patients (52.3%), lesions were localized in the lower extremities, in 13 (29.5%) on the upper limbs, in nine (20.5%) in the torso, and in six (13.6%) in the cervical-facial region (head), with evolution time ranging from 20 to 270 days (median = 60 days). Forty-one patients underwent MST testing and presented reactions ranging from 5 to 60 mm (median = 16 mm), with only one patient (2.3%) classified as a non-reactor (4 mm). The ELISA test was positive for 82.5% (33/40) of patients, and IIF was positive for 66.7% (26/39) of patients. Thirty-five patients (79.5%) acquired the infection in Rio de Janeiro and nine (20.5%) in other states of Brazil [three patients in Bahia (6.8%), two in Amazonas (4.5%), and one (2.3%) in each of the following states: Rondônia, Minas Gerais, Goiás, and Maranhão]. Forty-one patients were treated with meglumine antimoniate 5 mg Sb^5+^/kg/day, and three others received meglumine antimoniate 20 mg Sb^5+^/kg/day. Twenty-three patients (52.3%) were R to one course of treatment, and 21 (47.7%) were NR. However, all 21 patients healed after one to three additional (median = 1) courses of treatment. Seventeen of these patients showed lesion healing after further courses of 5 mg Sb^5+^/kg/day, including two patients who did not respond to 20 mg Sb^5+^/kg/day. Four other NR patients were cured after additional courses of amphotericin B (three) or pentamidine (one) (Figure 1). Table 1 shows a comparison of clinical and geographic data for the R and NR groups involved in the study. MST values were statistically higher in the R group (*p* = 0.048), and the NR group was more reactive to IIF as compared with the R group (*p* = 0.001).

**Table 1:** Comparison between clinical and geographic data of the responder and non-responder groups involved in the study.

Patient data	Responders (*n* = 23)	Non-responders (*n* = 21)
Gender		
Female	11 (47.8%)	7 (33.3%)
Male	12 (52.2%)	14 (66.7%)
Age (mean)		
Mean ± SD	36.04 ± 11.13	32.29 ± 13.37
Number of skin lesions		
Median (min–max)	1 (1–7)	1 (1–15)
Lesion location		
Lower extremity	10 (43.5%)	13 (61.9%)
Upper limbs	7 (30.4%)	6 (28.6%)
Head and cervical-facial	1 (4.3%)	5 (23.8%)
Torso	7 (30.4%)	2 (9.5%)
Lesion-evolution time (days)		
Median (min–max)	60 (30–150)	60 (20–270)
Number of treatments^a^		
Median (min–max)	1 (1–1)	2 (2–4)
MST		
Reactor	22 (100%)	18 (94.7%)
Non-reactor	–	1 (5.3%)
MST values (mm)^b^		
Median (min–max)	20 (7–60)	12 (4–27)
ELISA		
Reactor	16 (76.2%)	17 (89.5%)
Non-reactor	5 (23.8%)	2 (10.5%)
IIF^c^		
Reactor	9 (42.9%)	17 (94.4%)
Non-reactor	12 (57.1%)	1 (5.6%)
Region of Infection		
Rio de Janeiro	18 (78.3%)	17 (81%)
Other states	5 (21.7%)	4 (19%)

MST = Montenegro Skin Test; IIF = Indirect Immunofluorescence; RJ = Rio de Janeiro; OS = other states.

Mean ± SD = Mean and standard deviation.

*p* < 0.05 values are considered significant.

a *p *< 0.001 (Mann-Whitney U Test).

b *p* = 0.048 (Mann-Whitney U Test).

c *p* = 0.001 (Fisher's Exact Test).

### Phenetic analysis

**Figure 2:**
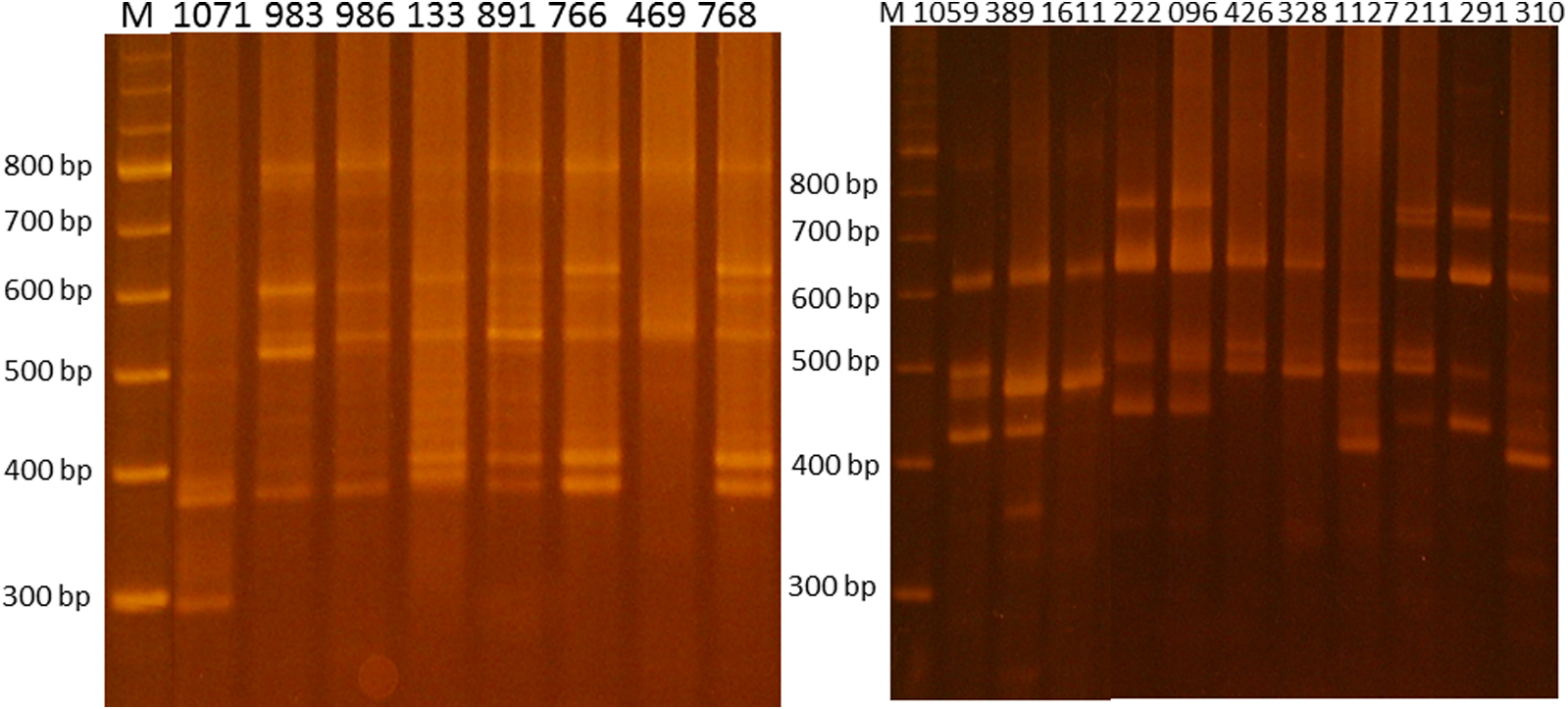
1.5% agarose gel electrophoresis showing representative LSSP-PCR profiles from parasites belonging to clusters I, II and III.

After specific PCR for the *L. braziliensis* complex, the diagnostic band of 750 bp was present in 100% of the *L. (V.) braziliensis* isolates (*n* = 44), as expected. Intra-population genetic variability was revealed by the polymorphic minicircle profiles obtained by the LSSP-PCR technique. Genetic profiles with different degrees of complexity were detected. Table 2 shows the 14 bands shared among the isolates. The *L. (V.) braziliensis* reference strain (MHOM/BR/75/M2903) was used as an internal control sample. In these experiments, as well as in others from our group [14], the reproducibility of the LSSP-PCR technique was confirmed when identical genetic profiles were observed in assays repeated at least three times under the same conditions. The same genetic profile was also observed when parasite DNA amplified directly from the biopsy of a cutaneous lesion was compared with DNA from promastigotes obtained after isolation in culture of a cutaneous lesion from the same patient (data not shown). Figure 2 shows representative LSSP-PCR profiles from strains belonging to clusters I, II and III.

**Table 2:** Data obtained from genetic analyses of all the 44 samples of *Leishmania (Viannia) braziliensis* studied.

Sample code^*^	Therapeutic outcome	Origin (state)	Shared bands	Similarity Index	Cluster
*L. braziliensis* reference	–	–	1, 7, 8, 9	0.93	II
1071	R	Rio de Janeiro	1, 7, 9, 10, 13	0.80	I
983	R	Rio de Janeiro	1, 5, 7, 10	1	I
986	R	Rio de Janeiro	1, 5, 7, 10	1	I
891	R	Rio de Janeiro	1, 5, 7, 9, 10	0.93	I
133	R	Rio de Janeiro	5, 7, 9, 10	0.89	I
766	R	Rio de Janeiro	1, 5, 6, 7, 9, 10	1	I
768	R	Rio de Janeiro	1, 5, 6, 7, 9, 10	1	I
1107	R	Rio de Janeiro	1, 7, 8, 10	0.93	II
811	R	Rio de Janeiro	1, 7, 8, 9, 10	0.93	II
475	NR	Rio de Janeiro	1, 7, 9	0.93	II
554	R	Rio de Janeiro	1, 7	1	II
345	NR	Rio de Janeiro	1, 7	1	II
952	NR	Rio de Janeiro	1, 7	1	II
378	NR	Rio de Janeiro	1, 7	1	II
1517	NR	Rio de Janeiro	1, 7	1	II
489	NR	Rio de Janeiro	1, 7	1	II
469	R	Rio de Janeiro	1, 4, 7	0.93	II
622	R	Goiás	1, 6, 7, 10	0.93	II
530	NR	Rio de Janeiro	1, 7, 10	1	II
1611	NR	Bahia	1, 7, 10	1	II
992	R	Rio de Janeiro	1, 4, 7, 10	0.93	II
145	NR	Rio de Janeiro	1, 4, 7, 9, 10	0.93	II
600	R	Rio de Janeiro	1, 7, 11	1	II
789	R	Rio de Janeiro	1, 7, 11	1	II
409	R	Rio de Janeiro	1, 2, 7, 11	0.93	II
501	R	Rio de Janeiro	1, 3, 7, 11	0.92	II
299	R	Rio de Janeiro	1, 4, 7, 8, 11	0.93	II
317	R	Rio de Janeiro	1, 4, 7, 11	0.93	II
426	NR	Rio de Janeiro	1, 7, 9, 10, 11	0.93	II
328	NR	Rio de Janeiro	1, 7, 10, 11	0.93	II
946	NR	Rondônia	1, 5, 7, 12	0.76	II
420	R	Minas Gerais	7, 10, 12, 14	0.93	III
1300	NR	Amazonas	7, 10, 12	1	III
389	NR	Maranhão	7, 10, 12	1	III
1453	R	Bahia	7, 10	1	III
1450	R	Bahia	7, 10	1	III
291	NR	Rio de Janeiro	4, 7, 10, 12	0.89	III
812	R	Amazonas	4, 7, 9, 12	0.93	III
211	NR	Rio de Janeiro	4, 7, 9, 10, 12	0.93	III
1059	NR	Rio de Janeiro	7, 9, 10, 12	0.91	III
222	NR	Rio de Janeiro	4, 7, 10, 11	1	III
96	NR	Rio de Janeiro	4, 7, 10, 11	1	III
310	NR	Rio de Janeiro	4, 7, 14	0.76	III
1127	NR	Rio de Janeiro	10, 13	0.72	III

^*^ Code of the Leishmania isolates.

R = responder group, NR = non-responder group (responder or non-responder to one course of treatment with meglumine antimoniate 5 mg Sb^5+^/kg/day or 20 mg Sb^5+^/kg/day).

**Figure 3:**
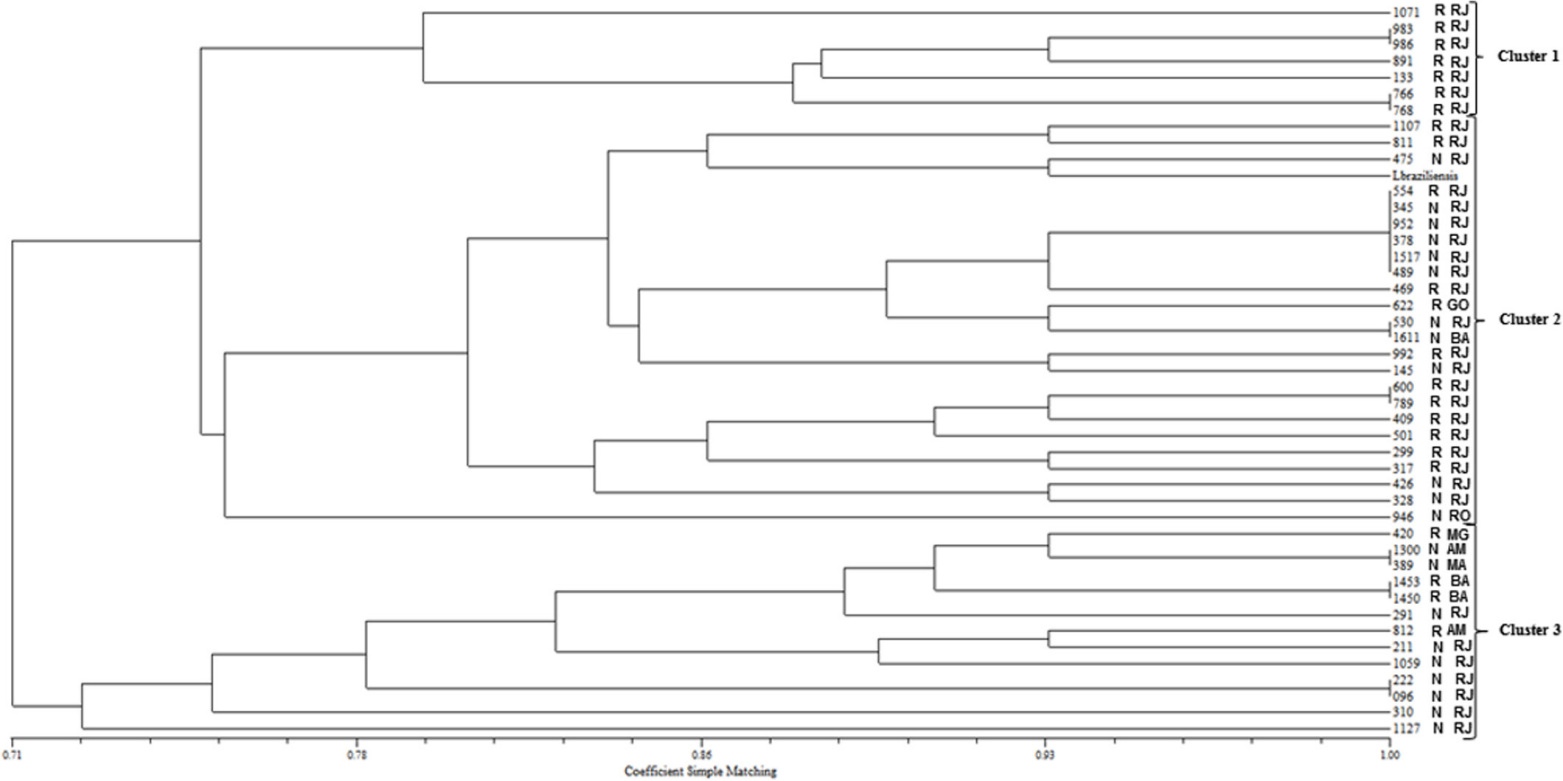
Phenetic analysis using the Simple Matching coefficient of similarity and the UPGMA algorithm. Three clusters (I, II and III) were identified. The *L. (V.) braziliensis* sample indicated in cluster II is the reference strain. R = responders; N = non-responders; RJ = Rio de Janeiro; GO = Goiás; BA = Bahia; RO = Rondônia; MG = Minas Gerais; AM = Amazonas; MA = Maranhão.

The isolates showed coefficients of similarity varying from 0.72 to 1.00 and were grouped into three main clusters (I, II, and III) through phenetic analysis (Table 2 and Figure 3). Cluster I was entirely composed of R patient samples from the State of Rio de Janeiro (total of seven samples). Four samples were found to be genetically identical, sharing all common characteristics (similarity index = 1.00) in this cluster. Cluster II grouped 24 isolates together with the *L. (V.) braziliensis* reference (MHOM/BR/75/M2903), of which 10 were found presenting a similarity index of 1.00. Most samples were grouped by treatment-response profile, as well as by the site of infection. Moreover, nine of 13 NR samples were grouped in cluster III, which included the majority of patient samples (six) from other states in Brazil, with all six isolates sharing 100% similarity, and all of them were grouped based on treatment response and infection locality. In total, 20 samples displayed 100% similarity, with samples 310, 1127, and 946 the most genetically differentiated and presenting similarity indices of 0.72, 0.76, and 0.76, respectively.

### Patient clinical data: inter- and intra-cluster evaluation

Patients with samples grouped in cluster II were 9 years older than those in cluster III and presented a lower frequency of lesions on the upper limbs as compared with those with samples grouped in cluster I. Cluster III included a higher number of patients reactive to the IIF test as compared with cluster I. All samples in cluster I were isolated from patients who reported infection in Rio de Janeiro and responded well to the first treatment course. Cluster II included more patient samples from Rio de Janeiro than cluster III, but these clusters did not differ regarding frequency of responders or even the number of treatments (Table 3). Statistical analysis showed significance when intra- and inter-cluster variables, including age, number of treatments, IIF, and region of infection, were compared (Table 3). Additionally, the Shapiro-Wilk normality test revealed that the variables, including number of treatments, MST (mm), number of lesions, and evolution time (days), escaped from normality and indicated non-rejection of normality for the variable “age”.

**Table 3:** Clinical and laboratory data of the 44 patients with cutaneous leishmaniasis studied. Intra- and inter-clusters division (numerical and categorical variables).

Patient data	Cluster I (*n* = 7)	Cluster II (*n* = 24)	Cluster III (*n* = 13)
Gender			
Female	3 (42.9%)	8 (33.3%)	7 (53.8%)
Male	4 (57.1%)	16 (66.7%)	6 (46.2%)
Age (mean)^a^			
Mean ± SD	33.29 ± 9.48	37.75 ± 12.16	28.31 ± 12.09
Number of skin lesions			
Median (min–max)	1 (1–1)	1 (1–15)	2 (1–7)
Lesion location			
Lower extremity	2 (28.6%)	15 (62.5%)	6 (46.2%)
Upper limbs^b^	5 (71.4%)	3 (12.5%)	5 (38.5%)
Head and cervical-facial	–	3 (12.5%)	3 (23.1%)
Torso	–	7 (29.2%)	2 (15.4%)
Lesion-evolution time (days)			
Median (min–max)	45 (30–90)	60 (20–180)	45 (30–270)
Number of treatments^c^			
Median (min–max)	1 (1–1)	1.50 (1–4)	2 (1–4)
MST			
Reactor	6 (100%)	24 (100%)	10 (90.9%)
Non-reactor	–	–	1 (9.1%)
MST values (mm)			
Median (min–max)	21 (7–60)	19 (7–57)	12 (4–27)
ELISA			
Reactor	4 (66.7%)	20 (90.9%)	9 (75%)
Non-reactor	2 (33.3%)	2 (9.1%)	3 (25%)
IIF^d^			
Reactor	2 (28.6%)	14 (66.7%)	10 (90.9%)
Non-reactor	5 (71.4%)	7 (33.3%)	1 (9.1%)
Response^e^			
Responder	7 (100%)	12 (50%)	4 (30.8%)
Non-responder	–	12 (50%)	9 (69.2%)
Region of Infection^f^			
Rio de Janeiro	7 (100%)	21 (87.5%)	7 (53.8%)
Other states	–	3 (12.5%)	6 (46.2%)

MST = Montenegro Skin Test; IIF = Indirect Immunofluorescence; RJ = Rio de Janeiro; OS = other states.

Mean ± SD = Mean and standard deviation.

*p *<  0.05 values are considered significant.

a (Clusters II and III) *p* = 0.030 (T-Test).

b (Clusters I and II) *p* = 0.006 (Fisher's Exact Test).

c (Clusters I and II) *p* = 0.048 (Mann-Whitney U Test) and (clusters I and III) *p* = 0.011 (Mann-Whitney U Test).

d (clusters I and III) *p* = 0.013 (Fisher's Exact Test).

e (Clusters I and II) *p* = 0.026 (Fisher's Exact Test) and (clusters I and III) *p* = 0.005 (Fisher's Exact Test).

f (Clusters II and III) *p* = 0.042 (Fisher's Exact Test).

Figure 1 shows the treatment regimen used and the therapeutic outcome of the 44 patients separated by clusters. Among patients from other states, only one patient in cluster III required a third treatment with amphotericin B, whereas the others were cured with one or two treatments of meglumine antimoniate. Regarding patients from Rio de Janeiro, two required an additional treatment with amphotericin B (one in cluster II and another in cluster III) and one with pentamidine (cluster II). All other patients from Rio de Janeiro were cured with one (cluster I) or four treatment courses (cluster II) of meglumine antimoniate. Interestingly, the parasite genotypes assembled in cluster I were all from R patients from Rio de Janeiro.

## Discussion

Pentavalent antimonials have been used to treat all clinical forms of leishmaniasis for the previous 70 years. Despite their efficacy, variability in responses has been reported, from clinical cure to the occurrence of therapeutic failures and relapses [17]. Studies reporting resistance to antimonials have been published [18,19]. The factors that lead to different clinical courses (spontaneous healing, lesion persistence, recurrences, and development of mucosal lesions) and the participation of parasite-virulence factors remain a challenge for researchers.

This study provided information on genetic polymorphisms in mitochondrial genomes (kDNA minicircles) from a panel of samples isolated from CL patients found to be R or NR to one course of treatment with meglumine antimoniate in Rio de Janeiro and other states in Brazil.

Genetic polymorphisms in *Leishmania* parasites can be used to determine possible correlations between different clinical forms, evolutionary patterns of the disease, and therapeutic responses [8,12,14]. In this context, molecular techniques are of fundamental importance to obtain the appropriate information when investigating the participation of the parasite and perhaps a specific genetic pattern in the therapeutic response of selected human CL cases. Although kDNA minicircles are not representative of the full genome, and no approach targeting drug-resistance genes was used here, genetic heterogeneity in *L. (V.) braziliensis* parasites recovered from cutaneous lesions was evident, and three phenetic clusters were revealed. A previous study reported that switches in kDNA-minicircle dominance indicate that factors other than the amplified chromosomal DNA cause minicircle switching [20]. Despite questions concerning distinct molecular clocks, polymorphisms are being detected in one or both nuclear and kinetoplast genomes and correlated with biological features [8].

Extra-nuclear genomes evolve faster than nuclear genes, making kDNA a good target for evaluating intraspecies differences and discriminating species that have diverged more recently [21]. The advantage in these cases is that most of the changes observed appear to involve a shift in the dominance or copy number of different minicircle classes. Therefore, polymorphisms can be easily detected in some cases. Genetic polymorphisms in variants of *Leishmania* *(Leishmania) amazonensis* selected in vitro for drug resistance have been reported [22]. The dominant minicircles in a resistant strain were found to pre-exist as minor conserved divergent classes in parental wild-type cells [20]. Polymorphisms in minicircles can emerge during infection with a single clone of the parasite, but can be reverted to the original pattern [8]. Such minicircle plasticity might be understood as a disadvantage, which potentially limits, in some cases, interpretation of the results.

Although no specific genotype could be associated with response or non-response to treatment, at least one group of *L. (V.) braziliensis* genotypes that circulates in Rio de Janeiro was discriminated in clusters I and III, showing phenotypes of good and poor responses to treatment, respectively. Therefore, we also established that parasites sharing all common genetic characteristics and belonging to the same phenetic cluster might be involved in different responses to treatment, as was the case in two patients from Rio de Janeiro (554 and 345). NR patients were prevalent in cluster III, given that most of them were isolated from Rio de Janeiro. Such results can be considered of clinical importance, because they allow genetic discrimination of distinct populations of *L. (V.) braziliensis* circulating in Rio de Janeiro and displaying different phenotypes.

Good response to antimony treatment in Rio de Janeiro might be explained by the genetic homogeneity observed in *L. (V.) braziliensis* [9,12]. In other regions, the heterogeneity of this species may hamper favorable treatment response [23,24]. However, in this study, the high percentage of genetic similarity (87%) found among the isolates from Rio de Janeiro and other states did not appear to influence therapeutic response, because we found patients in whom the isolated parasites displayed 100% genetic similarity and who showed different responses to treatment. Therapeutic response to pentavalent antimony is usually more diverse in other regions of Brazil, where different *Leishmania* spp. circulate, and greater genetic variability in *L. (V.) braziliensis* occurs [5,7,25]. However, four samples in cluster III and two in cluster II from patients infected in other states and treated at INI healed with treatment consisting of low-dose meglumine antimoniate. Such results suggested that even patients infected in other states in Brazil, and from whom genetically similar parasites were recovered, exhibited good response to treatment with meglumine antimoniate 5 mg Sb^5+^/kg/day. However, the NR group from Rio de Janeiro and other states included patients who required additional treatment with amphotericin B and pentamidine. Briefly, 81% of the NR patients (17 of 21) had their lesion healed after the second or third additional treatment with the low-dose regimen of meglumine antimoniate.

Patient age and lesion location(s) are among the factors that can interfere with the clinical course of the patient. Interestingly, we observed that the youngest group of patients placed in cluster III included a higher frequency of NR patients. Young age is considered to be the most solid predictor of treatment failure [26]. However, the increased frequency of lesions on the upper limbs as observed in cluster I was likely related to good therapeutic response, because lesions on the lower extremities have been associated with a prolonged therapeutic response [27].

It is also worth mentioning that patients presenting one cutaneous lesion, as observed in cluster I, might have influenced the good therapeutic response observed in this cluster [28]. Additionally, the statistical significance observed from clinical and laboratory data confirmed correlations between patients with poor response to treatment and reactivity to IIF tests (*p* = 0.013), especially when compared with cluster I, where all were R patients. A strong immune response to cutaneous leishmaniasis that favors ultimate full recovery is characterized by the activation of T helper (Th)_1_ response, which stimulates the production of interferon-γ, tumor necrosis factor-α and interleukin (IL)-2 cytokines, and induces low production of antibodies [29]. However, Th_2_ response is characterized by the secretion of IL-4, IL-10, and IL-13, which stimulate production of antibodies by B cells. This response is not efficient for infection control, and can cause susceptibility and persistence [30].

It is important to highlight that the results obtained by LSSP-PCR can be considered to have clinical and epidemiological relevance. Further studies related to in vitro sensitivity of promastigotes and amastigotes to pentavalent antimonials are necessary to corroborate these findings.
